# A cluster randomized controlled trial of an electronic decision-support system to enhance antenatal care services in pregnancy at primary healthcare level in Telangana, India: trial protocol

**DOI:** 10.1186/s12884-022-05249-y

**Published:** 2023-01-26

**Authors:** Sailesh Mohan, Monica Chaudhry, Ona McCarthy, Prashant Jarhyan, Clara Calvert, Devraj Jindal, Rajani Shakya, Emma Radovich, Dimple Kondal, Loveday Penn-Kekana, Kalpana Basany, Ambuj Roy, Nikhil Tandon, Abha Shrestha, Abha Shrestha, Biraj Karmacharya, John Cairns, Pablo Perel, Oona M. R. Campbell, Dorairaj Prabhakaran

**Affiliations:** 1 Public Health Foundation of India (PHFI), Plot 47, Sector 44, Gurugram, Haryana 122002 India; 2 Centre for Chronic Disease Control (CCDC), Safdarjung Development Area, C-1/52, Second Floor, Delhi, 110016 India; 3 London School of Hygiene & Tropical Medicine, Keppel St, London, WC1E 7HT UK; 4 Old Medical School, Usher Institute, University of Edinburgh, Teviot Place, Edinburgh, EH8 9AG UK; 5 Dhulikhel Hospital, Kathmandu University, JG8X+P54, Dhulikhel, 45200 Nepal; 6 SHARE (Sci Health Allied Res Education), MediCiti Institute of Medical Sciences Campus, Medchal-Malkajgiri, Hyderabad, Telangana 501401 India; 7 All India Institute of Medical Sciences, Sri Aurobindo Marg, Ansari Nagar, New Delhi, Delhi, 110029 India

**Keywords:** Mobile health, Digital health, Electronic decision-support system, Clinical decision-support system, Antenatal care, Maternal health services, Health system redesign, Pregnancy Induced Hypertension, Gestational Diabetes Mellitus, Anaemia in pregnancy, Cluster randomized trial

## Abstract

**Background:**

India contributes 15% of the total global maternal mortality burden. An increasing proportion of these deaths are due to Pregnancy Induced Hypertension (PIH), Gestational Diabetes Mellitus (GDM), and anaemia. This study aims to evaluate the effectiveness of a tablet-based electronic decision-support system (EDSS) to enhance routine antenatal care (ANC) and improve the screening and management of PIH, GDM, and anaemia in pregnancy in primary healthcare facilities of Telangana, India. The EDSS will work at two levels of primary health facilities and is customized for three cadres of healthcare providers – Auxiliary Nurse Midwifes (ANMs), staff nurses, and physicians (Medical Officers).

**Methods:**

This will be a cluster randomized controlled trial involving 66 clusters with a total of 1320 women in both the intervention and control arms. Each cluster will include three health facilities—one Primary Health Centre (PHC) and two linked sub-centers (SC). In the facilities under the intervention arm, ANMs, staff nurses, and Medical Officers will use the EDSS while providing ANC for all pregnant women. Facilities in the control arm will continue to provide ANC services using the existing standard of care in Telangana. The primary outcome is ANC quality, measured as provision of a composite of four selected ANC components (measurement of blood pressure, blood glucose, hemoglobin levels, and conducting a urinary dipstick test) by the healthcare providers per visit, observed over two visits. Trained field research staff will collect outcome data via an observation checklist.

**Discussion:**

To our knowledge, this is the first trial in India to evaluate an EDSS, targeted to enhance the quality of ANC and improve the screening and management of PIH, GDM, and anaemia, for multiple levels of health facilities and several cadres of healthcare providers. If effective, insights from the trial on the feasibility and cost of implementing the EDSS can inform potential national scale-up. Lessons learned from this trial will also inform recommendations for designing and upscaling similar mHealth interventions in other low and middle-income countries.

Trial Registration.

ClinicalTrials.gov, NCT03700034, registered 9 Oct 2018, https://www.clinicaltrials.gov/ct2/show/NCT03700034

CTRI, CTRI/2019/01/016857, registered on 3 Mar 2019, http://www.ctri.nic.in/Clinicaltrials/pdf_generate.php?trialid=28627&EncHid=&modid=&compid=%27,%2728627det%27

**Supplementary Information:**

The online version contains supplementary material available at 10.1186/s12884-022-05249-y.

## Contributions to the literature

- The first trial in India to evaluate the effectiveness of an electronic decision-support system (mIRA EDSS) targeted at enhancing the quality of ANC, designed for multiple levels of primary health facilities and cadres of healthcare providers as its users.
- The mIRA EDSS has been co-designed with international and national subject experts, regional and local stakeholders, including state and district health officials and its targeted users (the healthcare providers)

## Background

India has significantly reduced its maternal mortality ratio (MMR) by 70% in a span of just two decades [1, 2]. However, there is still a long way to go to achieve the Sustainable Development Goal of reducing the MMR to 70 deaths per 100 000 live births by 2030 [3]. Despite this reduction, India still accounts for 15% of the global maternal mortality burden [4, 5]. The country is also undergoing an obstetric transition [6] with indirect obstetric causes, especially Non-communicable Diseases (NCDs), emerging as significant contributors to maternal mortality [7–9]. Recent studies report hypertension to be the most common NCD amongst pregnant women in India [10, 11], while the prevalence of Gestational Diabetes Mellitus (GDM) is also rising [12, 13]*.* Furthermore, over half of women in India are anemic [14]*.*

Antenatal care (ANC) plays a critical role in the early detection and prompt management of high-risk pregnancies, including timely referral to specialist services when required [15]. Coverage is high, but the quality of antenatal care delivered across Indian states and different socioeconomic groups is highly variable [16, 17]. This gap needs to be addressed by standardizing the quality of ANC delivered in public health facilities, which are used by a large proportion of rural, vulnerable populations. The ubiquitous use of mobile phone technology in India [18], including in healthcare settings, offers a potential pathway to address the gaps and improve the quality of antenatal care [19–21].

Decision-support systems commonly feature as a part of the mHealth interventions tested in healthcare. Various decision-support systems have been implemented globally, but there is dearth of literature when it comes their use in enhancing ANC and improving the management of NCDs in pregnant women in low-and middle-income countries (LMICs) [22, 23]*.* Only a few mHealth interventions with a decision-support system have been evaluated for ANC in India [24–28], and none thus far, has targeted multiple healthcare providers at different levels of the primary healthcare system. Therefore, the goal of this study is to address this critical gap in the provision of quality ANC at the primary healthcare level in India, by co-designing/developing and evaluating the effectiveness of a tablet-based, electronic decision-support system (EDSS). This mHealth integrated model of pregnancy-induced hypertension, gestational diabetes mellitus, anaemia, and antenatal care (mIRA EDSS) to improve the quality of ANC will be evaluated through a cluster randomized controlled trial.

## Methods

### Aims and objectives

The overall aim of this study is to evaluate the effectiveness of the mIRA EDSS in improving the quality of ANC in India. The mIRA EDSS comprises a tablet-based android application to deliver guideline-based routine ANC along with screening for, and subsequent management of, pregnancy induced hypertension (PIH), GDM, and anaemia.

The primary objective is to evaluate the effectiveness of the mIRA EDSS intervention in improving the quality of ANC in the intervention arm compared to standard ANC to the control arm, assessed by changes in the provision of four selected ANC components (the measurement and recording of blood pressure; and the conduct of: a blood glucose test, a urinary dipstick test, and a hemoglobin test).

The secondary objectives are to evaluate whether the mIRA EDSS improves the following components of ANC in the intervention arm as compared to  the control arm:

1. Provider identification of PIH, GDM, and severe anaemia, and their communication about this to the participants (pregnant women)
2. The quality of provider communication about pregnancy symptoms and response
3. Provider counselling on danger signs
4. Provider delivery of a comprehensive suite of quality ANC components

### Study design and setting

**Fig. 1:**
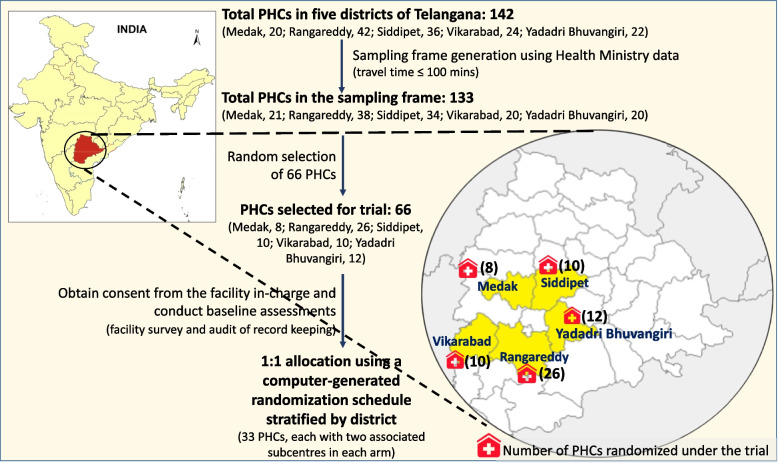
Study setting and randomization of facilities Legend: Source for map image: igis map (https://map.igismap.com/converter), an open source online platform

The study is a cluster randomized controlled trial with a 1:1 allocation ratio (stratified by district), evaluating the effectiveness and cost-effectiveness of mIRA EDSS. The trial will be conducted in government healthcare facilities [primary healthcare centres (PHCs) and their associated subcentres (SCs)] in five districts of Telangana state in Southern India (Fig. 1). In Telangana, 95% of women received ANC from a skilled provider [29], but care was inadequate for more than 50% of women [17], about 60% of the pregnancies were reported as high-risk [30], and more than half the pregnant women were anemic [31]. GDM is the fourth most common cause of a high-risk pregnancy in the state [30, 32]. We selected five districts: Medak, Rangareddy, Siddipet, Vikarabad, and Yadadri Bhuvangiri around the state capital (Hyderabad), including those with a low levels for provision of adequate ANC (Rangareddy, 24.7%, and Medak, 25.1%) [33].

### Intervention and its co-design

**Fig. 2:**
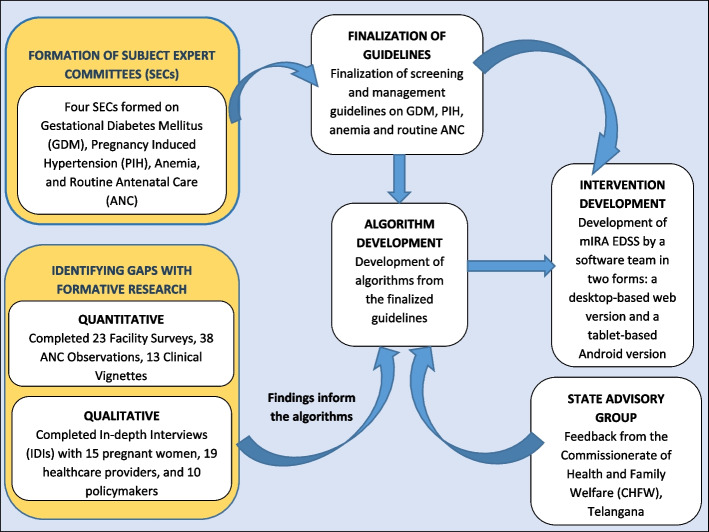
Co-designing of the study intervention

The mIRA EDSS was developed based on a robust formative research process with the target population, and was co-designed with multi-stakeholder engagement involving international and national experts, state and district health officials, and the healthcare providers (targeted users). This co-designing approach resulted in an EDSS that provides automated decision-support prompts of guideline-recommended processes of ANC (e.g. screening for anaemia, treatment, and counseling), tailored to each pregnant woman’s individual needs (i.e. her obstetric history/vital signs/abdominal examination and results of diagnostic tests). It also provides a checklist of components specific to each ANC visit and enables regular longitudinal monitoring of the pregnant women. Briefly, the development process for the intervention comprised of the following phases (Fig. 2):

1. *Identifying gaps:* Mixed-methods formative research was conducted in 23 facilities to assess the health infrastructure and ANC service provision in the SCs and PHCs of Telangana and among pregnant women, health care providers, administrators and state health officials. The findings revealed that most health facilities have adequate infrastructure and equipment to support the mIRA EDSS. However, important components of ANC like ‘history taking’ and ‘counseling’ were performed sub-optimally at all the facilities. Also, some elements of the physical examination (assessment of oedema, pallor, pulse rate, respiratory rate, and jaundice) and some investigations (urine protein, urine glucose, and blood glucose) were frequently missed, underlining the scope and need for improvements.
2. *Formation of subject expert committees and finalization of guidelines*: Four Subject Expert Committees (one each for PIH, GDM, anaemia, and ANC), comprising of national and international experts provided input into the guidelines used to prepare the clinical algorithms.
3. *Development of the clinical algorithms*: The guidelines were used to create algorithms for decision-making and management of the aforementioned conditions for all stages of pregnancy.
4. *Adaptation of clinical algorithms for the local context*: The Commissionerate of Health & Family Welfare (CHFW), Telangana provided feedback on the algorithms during the development phase, which was used to tailor them to the state’s needs and priorities.
5. *Development of the mHealth application i.e., mIRA EDSS*: The mIRA EDSS was developed by a software company, Quad One Technologies Pvt Ltd. as a desktop-based web version and a tablet-based Android version. These two versions incorporated a rule engine (based on the developed clinical algorithms), which formed the backend, and; an electronic data-capture form (the user interface) which formed the frontend.

The mIRA EDSS includes the following components – (i) a user interface; (ii) a patient database (iii) algorithms to screen symptoms, signs, and clinical parameters recorded by the healthcare providers for high-risk conditions in pregnancy and for routine ANC; (iv) recommendations on management and referral according to the stage of pregnancy and the high-risk condition(s) detected. The mIRA EDSS guides the healthcare provider to provide key elements of ANC, alerts them to any danger signs identified by the clinical algorithms, and provides suggested recommendations. The recommendations presented by the mIRA EDSS for the MO will be different than the ones for ANMs and staff nurses, since, the former can prescribe medicines, conduct certain examinations, and undertake certain management steps that the ANMs and staff nurses are neither trained nor authorized to perform. The items recorded on the EDSS include medical history, findings from clinical and physical examinations, counseling and preventive measures provided.

### Intervention delivery

**Fig. 3:**
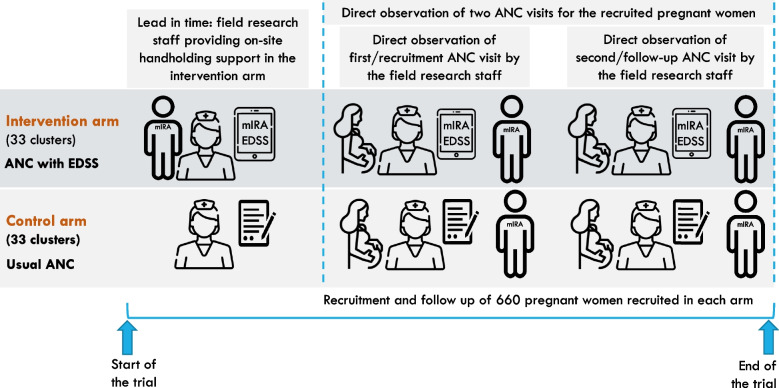
Schema for evaluating the effect of mIRA EDSS

In the intervention arm, ANC will be provided to all pregnant women by the ANMs, staff nurses, or Medical Officers (MOs) using the mIRA EDSS. These frontline healthcare providers will be trained to use the mIRA EDSS and will also be provided with refresher training on the guideline-recommended processes of ANC. This training will differ for MOs and the ANMs/staff nurses, so as to enable them to use the mIRA application in the provision of ANC within the limits of their authorities. The trial’s schema is presented in Fig. 3.

Facilities randomized to the control arm will continue to provide ANC services as per the existing standards of care. Evidence-based guidelines in the form of posters/pamphlets on the current national and state guidelines pertaining to the screening and management of PIH, GDM, and anaemia, and routine ANC procedures will be provided to all facilities in both the study arms.

### Facility selection

A list of all PHCs and their associated SCs was obtained from the Commissionerate of Health and Family Welfare (CHFW), Telangana, India. The eligible health facilities (PHCs and their SCs) that fulfilled the inclusion criteria mentioned below formed the sampling frame.

#### *Inclusion criteria*:

- PHCs with at least two associated SCs and having at least 40 first ANC visits (registrations) per year
- PHCs located within 150 min travel time from the CHFW, Hyderabad (Fig. 1)
- Facilities with adequate infrastructure and human resources to support the intervention (assessed via phone survey by research staff): electricity to charge tablets; reported availability of at least one functioning blood pressure apparatus; adequate human resources [at least one ANM at the SC and one staff nurse and one Medical Officer (MO) at the PHC]
- Facilities situated in a locality with self-reported reliable/good internet connectivity

#### Exclusion criteria

Health facilities in which the facility in-charge states a strong reason for not implementing the intervention at the facility will not be selected. Examples of reasons (non-exhaustive) that would exclude the facility are: refusal to participate because of workload concerns; ANM turnover; availability of electricity for less than two hours a day; providers not fluent in the two languages (English and Telugu) in which mIRA is offered.

The project research staff will first conduct a phone survey to assess the eligibility of health facilities for inclusion into the trial. During the phone survey, the research staff will brief the facility in-charges about the trial, and arrange for an in-person visit to obtain their written informed consent. The phone survey will only be conducted at the PHC level. HMIS data on the associated SCs will be used to select two SCs with the highest number of ANC visits. Facilities that do not have sufficient capacity with respect to the above mentioned criteria will be replaced by another facility from the sampling frame.

### Recruitment of participants (pregnant women)

After randomization and completion of the lead-in time at the facilities in the intervention arm (described later), field research staff stationed at each trial facility (in both the intervention and control facilities) will recruit eligible pregnant women. When a pregnant woman visits the health facility for an ANC consultation, the healthcare provider (ANM at the SC, and Staff Nurse or the MO at the PHC) will brief her about the research study and ask if she is interested in participating. If so, the healthcare provider will introduce her to the field research staff from the project. The field research staff will determine her eligibility, based on the inclusion and exclusion criteria below, using a paper-based checklist:

#### Inclusion criteria

- Pregnant women aged 18 years or above
- Pregnant women visiting a trial facility up to the end of the 28th week of gestation
- Pregnant women who are planning to remain within the five study districts until at least one-month postpartum OR women whose mothers reside in the selected districts

#### Exclusion criteria

- Women coming to the trial facility for a non-routine ANC visit (for example, to get a laboratory investigation, or to collect a report or her medicine)

**Fig. 4:**
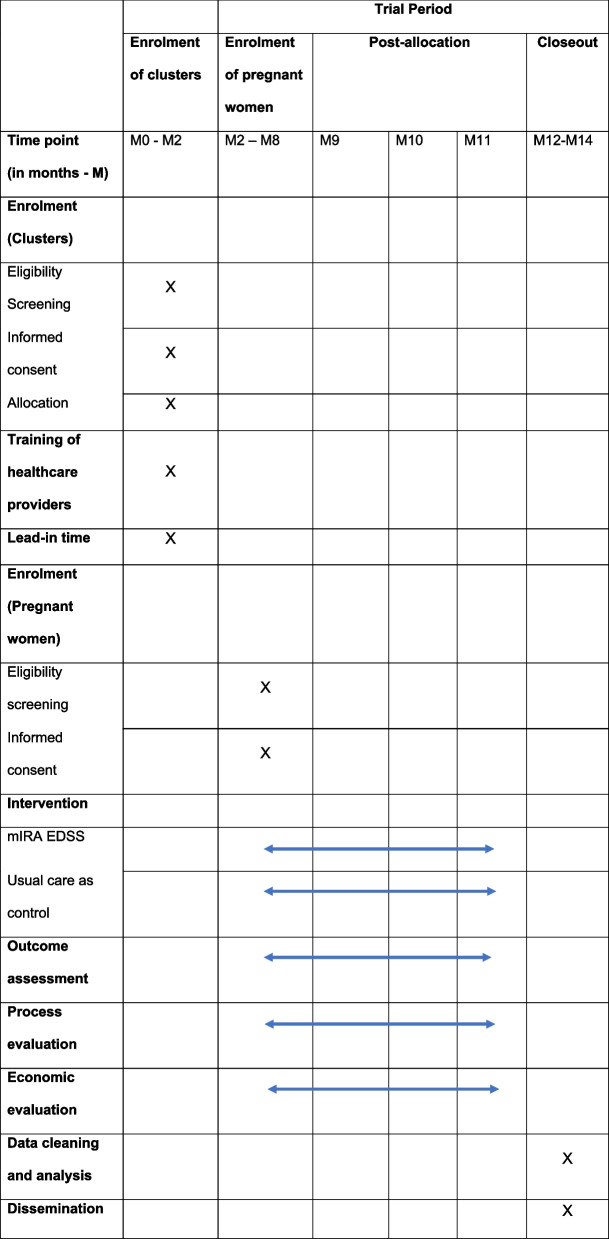
Schedule of enrolment, intervention allocation and assessment using Standard Protocol Items: Recommendations for Interventional Trials (SPIRIT) figure for study protocols

Once the field research staff confirms the pregnant woman’s eligibility, she will be provided with a participant information sheet (PIS) (Supplementary file 1) and the opportunity to read it (or have it to read to her if she is not literate), ask questions and consider participation. If she chooses to participate, the field research staff will provide her the informed consent form (ICF) (Supplementary file 2), ask her to carefully read and consider all the information listed on it, before she provides signed informed consent. A copy of the PIS and ICF will be given to each pregnant woman or her representative to keep. Women will be informed that they will be observed twice and that they will also be contacted by telephone. The schedule of enrolment, intervention allocation and assessment has been described using the Standard Protocol Items: Recommendations for Interventional Trials (SPIRIT) in Fig. 4.

### Cluster randomization

Sixty-six clusters will be randomly assigned to either the control or intervention group with a 1:1 allocation ratio via a computer-generated randomization schedule stratified by district (33 clusters in each arm). Randomization units will be clusters of three health facilities–a PHC and two linked SCs. Cluster randomization was preferred over the individual randomization to avoid contamination among the participants (pregnant women). Randomization will be performed by the trial statistician, who is not involved in the day to day running of the trial. Covariate constrained randomization will be used to balance the arms on the following baseline covariates: presence of a lab and lab technician (yes/no); more than three staff providing ANC (yes/no); facility type (PHCs that are always open/PHCs that are not).

### Sample size

We have designed this trial to detect a 12.5% relative difference in the mean number of ANC components measured and recorded over the two visits (eight total) between the control and the intervention (control mean = 4, intervention mean = 4.5, an absolute difference of 0.5). This difference is based on 1) the mean number of ANC components measured in the formative phase, which was 1.95 (standard deviation 0.9) for a single visit (doubled for the sample size calculation to account for the two-visit primary outcome) and 2) a pragmatic decision regarding the time and resources available for recruitment. Using an intra-cluster correlation coefficient (ICC) of 0.05 [34] and assuming an average cluster size of 20 women, 52 clusters, 1,040 pregnant women (520 in each arm) will have 90% power to detect a 12.5% relative increase in the mean number of ANC components over the two visits in the intervention arm compared to the control arm (control mean = 4.0, intervention mean = 4.5). Allowing for a 20% loss to follow-up, we will enroll 1,320 women (660 in each arm) across 66 clusters.

### Blinding

Because of the nature of this intervention, pregnant women, healthcare providers, and field research staff collecting the outcome data will be aware of allocation to the intervention or control arm, precluding blinding. However, adequate measures will be taken to avoid possible bias by the field research staff. The field research staff stationed at the facilities in the intervention arm during the lead-in period (an intensive support period described later) will be switched to the control arm before recruitment of women starts. The field research staff will also be trained to reduce the risk of observer bias while conducting the ANC observations. The team analyzing the data and interpreting the trial results will be masked to the study arm until all analyses outlined in the statistical analysis plan are completed.

## Outcomes

### Primary outcome

The primary outcome is the mean number of four selected ANC components delivered by the healthcare providers per visit, observed over two visits- the trial enrolment visit and the next routine ANC appointment (8 components total). The four selected components are: 1) the measurement and recording of blood pressure; and the performance of 2) blood glucose, 3) urinary dipstick, and 4) hemoglobin tests.

### Secondary outcomes

The secondary outcomes are the:

1. Mean number of the four ANC components observed at the enrolment visit.
2. Mean number of the following symptoms discussed with participants (either by the provider asking or the woman mentioning), observed over the two visits: nausea, vomiting, vaginal bleeding, severe headache, decreased or absent fetal movement, severe abdominal pain, and blurred vision.
3. Proportion of providers who took the appropriate action per participant (as recommended by the mIRA EDSS) in response to the aforementioned symptoms (vomiting, vaginal bleeding, severe headache, decreased or absent fetal movement, severe abdominal pain, blurred vision) or reported clinical parameters indicative of PIH, GDM, or severe anaemia.
4. Mean number of the danger signs (severe vomiting, vaginal bleeding, severe headache, decreased or no fetal movement, severe abdominal pain, blurred vision) mentioned to each participant by the healthcare provider for which she is advised to return for help.
5. Proportion of participants with clinical parameters indicative of PIH (SBP ≥ 140 mmHg and DBP ≥ 90 mmHg), GDM (venous blood glucose: fasting > 92 mg/dL, 1 h OGTT > 180 mg/dL, 2 h OGTT > 153–199 mg/dL; glucometer test values: fasting > 92 mg/dL, 1 h OGTT > 198 mg/dL, 2 h OGTT > 168–219 mg/dL) or severe anaemia (< 7 g/dL).
6. Proportion of participants who were told by the provider that they had PIH, GDM or severe anaemia.
7. Mean number of quality ANC components delivered in the enrolment visit. The components (n = 20) are as follows:
  tests completed: blood pressure; blood glucose; urinary dipstick; hemoglobin tests;
  symptom check: nausea; vomiting; vaginal bleeding; severe headache; decreased or absent fetal movement; severe abdominal pain; blurred vision;
  warning about: severe vomiting; vaginal bleeding; severe headache; decreased or no fetal movement; severe abdominal
  pain; blurred vision;
  enquiring about mental health;
  writing on the woman-held Mother and Child Protection card.

### Intervention training, implementation and monitoring

#### Training

The healthcare providers (ANMs, Staff Nurses, and MOs) at the facilities in the intervention arm will be trained in a 3-day workshop, which will focus on: how to use the mIRA EDSS; its hardware and software functionality; how the mIRA EDSS uses algorithms to improve adherence to existing ANC guidelines, and troubleshooting of potential glitches. This workshop will also provide training on the routine ANC procedures which will be a part of the clinical workflow of the mIRA EDSS. The sessions will be delivered by trained research staff and will also be attended by the Program Officers who have a supervisory role for the health facilities at the district level. All healthcare providers at the facilities in the intervention arm will receive a manual for operating the mIRA EDSS and for troubleshooting.

During the training workshop, the mIRA EDSS will be installed on the pre-existing tablets at the PHCs and SCs, and one additional tablet with the pre-installed mIRA EDSS will be provided to each of the PHCs (but not SCs) in the intervention arm.

The training program will be evaluated to assess: a) the healthcare providers’ ability to perform the key functions using the mIRA EDSS intervention; b) their responses to and understanding of the mIRA EDSS intervention following the training; and c) whether further training or additional support in using the mIRA EDSS intervention is needed. The healthcare providers with low scores will receive more intensive support during the lead-in time and will be trained again (online) to use the EDSS application.

#### Implementation

Healthcare providers at the facilities in the intervention arm will be given a lead-in time of 20 days following completion of the training, during which they will be provided with on-site support while using the mIRA EDSS to provide ANC. During this time, any errors encountered with the mIRA EDSS will be corrected and updates will be released. After the lead-in time, and the installation of the final version of the mIRA EDSS in the tablets being used in the intervention arm, the healthcare providers will be expected to use the mIRA EDSS to conduct ANC consultations for all pregnant women who visit the facilities. Field research staff will be stationed at the facilities in the intervention arm on rotation during the recruitment period and will conduct monthly monitoring visits to all facilities during the follow-up period.

#### Monitoring

The intervention will be monitored at frequent intervals from the start of implementation after the training, until the conclusion of the trial. Monitoring will consist of in-person monthly visits conducted by field research staff to the facilities in the intervention arm. During these visits to the facilities in the intervention arm, field research staff will fill a monitoring checklist to record hardware and mIRA EDSS software availability and functionality, identify newly posted staff in need of training in the mIRA EDSS, and record indicators of utilization for the process evaluation. Field research staff will document any technical problems with the tablet hardware or mIRA EDSS software and attempt to resolve them during the monitoring visit. Field research staff will explain any new changes made to the application, and ensure that the latest version is downloaded on the tablets. They will be instructed and trained not to intervene if tablets are not being used during ANC consultations and will not provide support if asked for help while conducting an ANC visit observation (but can help after the observation is over).

We will also conduct remote monitoring of mIRA EDSS usage data. Data from the mIRA EDSS will be used to assess the utilization of the intervention by frontline healthcare workers via timing and duration of logins, and the number of mIRA EDSS entries on a given date. The frequency of data upload to the server will be documented. GPS coordinates of the tablet during application use will be used to assess whether data entry is occurring at the facility site. This data will not be shared with the field research staff. Data on the weekly ratio of entries saved vs submitted, the proportion of applicable input fields completed, the proportion of mIRA EDSS recommendations accepted (vs ‘reject/modify’), and the proportion of records prompting a test with the test results recorded in the application will be used to assess the quality of mIRA EDSS utilization and intervention fidelity. We will document additional data on technical support, such as events of application crashes and other technical errors reported to designated mIRA research staff, time taken to resolve queries, or the number of requests received from healthcare providers for help with data editing in the application. This activity will not be used for course correction, beyond maintenance and technical support, during the trial’s implementation period.

### Data collection

**Fig. 5:**
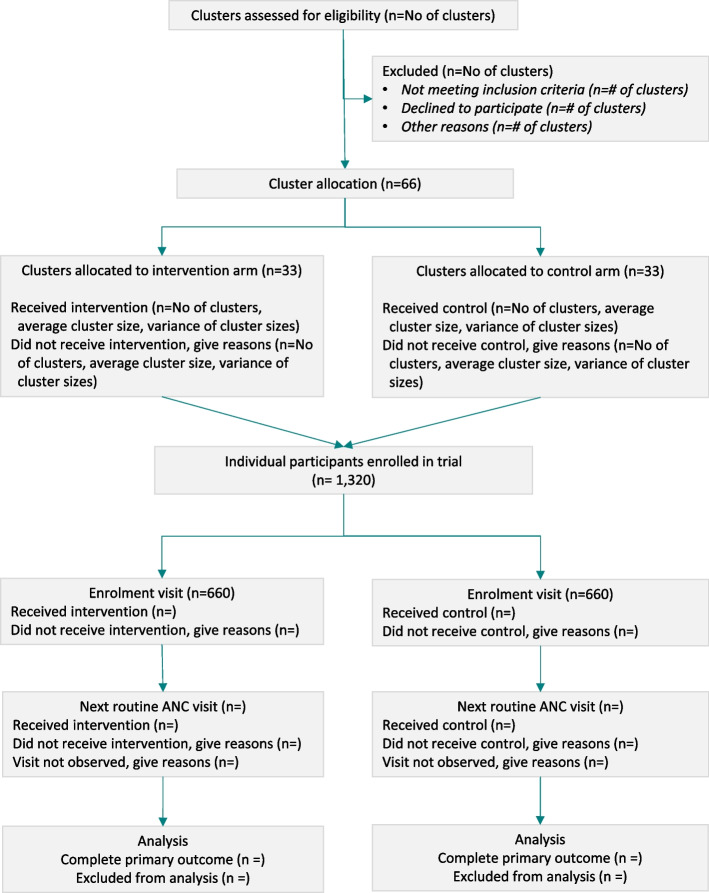
CONSORT Trial flow diagram

Field research staff stationed at or visiting the facilities will conduct ANC observations using an ANC observation checklist to collect the trial outcome data for the women who consented to participate in the trial in intervention and control facilities. This paper-based ANC checklist will be used to record the components of ANC completed by the health care provider during a consultation with pregnant women recruited under the trial. The first ANC observation will be conducted on the day of recruitment (the ‘enrolment visit’). The field research staff will collect contact details from the woman during the enrolment visit and will telephone the woman one week prior to her next scheduled routine ANC visit to confirm her appointment. Field research staff will then make sure to be present at the facility for the pregnant woman’s visit to conduct the second ANC observation. Throughout this time, all information will also be collected from paper-based records such as the ANC, high risk, and referral registers, and the Mother and Child Protection (MCP) cards. All trial outcome data will be collected via the ANC observation checklist (Supplementary file 3) & MCP cards to ensure comparability between arms. A summary of trial outcomes’ data collection, tools, sources and period of data collection has been provided in Table 1, and the CONSORT trial flow diagram is presented in Fig. 5.

**Table 1:** Data collection and source

Outcome	Data source	Time
The mean number of the four selected ANC components completed per visit over two visits (8 components) *(primary outcome)*	ANC observation tool	At enrolment visit and next routine ANC visit
The mean number of the four selected ANC components completed at the enrolment visit *(secondary outcome #1)*	ANC observation tool	At enrolment visit
The mean number of symptoms discussed either by the provider asking or the woman volunteering (nausea, vomiting, vaginal bleeding, severe headache, decreased or absent fetal movement, severe abdominal pain, blurred vision) observed over the two visits (*secondary outcome #2*)	ANC observation tool	At enrolment visit and next routine ANC visit
(among participants who said they were experiencing vomiting, vaginal bleeding, severe headache, decreased or absent fetal movement, severe abdominal pain or blurred vision or who were told that they had hypertension in pregnancy, GDM or anaemia) the action taken by the provider in response *(secondary outcome #3)*	ANC observation tool & MCP card	At enrolment visit and next routine ANC visit
The mean number of danger signs the provider tells the women she should return for help (*secondary outcome #4*)	ANC observation tool	At enrolment visit and next routine ANC visit
The proportion of participants were* told* by the provider that they had hypertension in pregnancy, GDM or severe anaemia (*secondary outcome #5*)	ANC observation tool	At enrolment visit and next routine ANC visit
proportion of participants for whom there was a recording of the diagnostic parameters of hypertension, GDM or severe anaemia (*secondary outcome #6*)	MCP card	At enrolment visit and next routine ANC visit
In the enrolment visit, the mean number of 20 components of quality antenatal care delivered *(secondary outcome #7)*	ANC observation tool	At enrolment visit

### Statistical methods

The primary analyses will be conducted according to randomised arm of the facility in which pregnant women were recruited. The continuous primary outcome (i.e. mean number of the four selected components of ANC observed over two visits) will be analysed at the individual level using Generalised estimating equation (GEE) analysis with identity-link function. GEE assumes that observations within the same clusters may be correlated. For the analysis of cluster randomized trials, it is usual to assume that the correlation matrix is exchangeable, meaning that observations on participants in different clusters are uncorrelated, while observations on individuals in the same cluster all have the same correlation coefficient ρ.

We will adjust the primary analysis regression for the following pre-specified baseline covariates likely to be associated with the outcome to improve the efficiency of the analysis and avoid chance imbalances [35]: gestational age at enrolment, number of previous ANC visits and scheduled caste or tribe.

For categorical secondary endpoint analyses i.e., the proportion of pregnant women who were told by the provider that they had hypertension in pregnancy, GDM or severe anaemia and proportion of pregnant women for whom there was a recording of the diagnostic parameters of hypertension, GDM or severe anaemia, the relative risk for these secondary outcome(s) between the mIRA EDSS and standard care group will be analysed at the individual level using GEE analysis with logarithmic-link function.

Recognising that the trial is not powered to detect effect differences in subgroups, we will conduct exploratory subgroup analyses for the primary outcome to determine if the intervention effect varies by the following pre-specified subgroups: age (split at the median); parity (0/1 or more); caste or tribe (yes/no); highest education level completed (completed primary or above/other); EDSS use (used for entire ANC consultation/not used for entire consultation) and gestational age at first visit (< 10 week/10 week or above). Within the pre-specified subgroups, we will assess heterogeneity of treatment effect with a test for interaction [5–9]. Interaction test p-values will be presented but will be interpreted with caution, due to the exploratory nature, the multiple tests performed and the low power of the interaction test. We will estimate beta coefficients along with 95% CIs for each subgroup. As this is an exploratory analysis of potentially influential characteristics, we will not hypothesise effect directions.

The primary outcome data with missing values will be handled under two different assumptions. First, the primary outcome will be considered as complete if it is confirmed that the participant attended the facility for the second visit. Data missing for both of these visits will be assumed and coded as component ‘not done’. Second, the primary outcome will not be considered as complete if the second visit data is missing and it is confirmed that the participant did not attend for this visit. In this case, participants’ data will not be included in the primary analysis.

### Process evaluation

A mixed-methods process evaluation will be nested within the trial assessing intervention effectiveness. The overall aim of the process evaluation is to generate evidence to inform the interpretation of the outcomes of the main trial, and to further understanding of the theories underpinning mIRA EDSS quality improvement interventions.

As part of the parallel mixed-method process evaluation [36], data for quantitative and qualitative analyses will be collected at multiple time points throughout the trial. Quantitative data collection methods will include ongoing routine monitoring from all sites. A baseline facility survey will be conducted for facilities in both trial arms to examine the role of structural quality and organizational factors in moderating the intervention process and effects. Monitoring data from in-person research staff visits to the facilities in the intervention arm and backend data from the mIRA EDSS will be used to evaluate the frequency and quality of use of mIRA. As mIRA will be implemented alongside existing paper-based record-keeping systems, we will conduct an audit of record-keeping practices to examine change in completeness and accuracy of the ANC register and MCP cards before and after mIRA EDSS implementation. Qualitative data collection methods will include regular debriefing meetings and field notes from field research staff involved in the trial implementation and end-line in-depth interviews with selected healthcare providers who used the mIRA EDSS; policymakers and research staff involved in the implementation of the intervention.

### Economic evaluation

We will conduct an economic evaluation to estimate the additional costs of the intervention relative to the value of the improved health outcomes achieved by calculating the additional cost of the intervention and the use of resources for providing ANC in the intervention and control arms.

The cost of the enhanced ANC intervention includes the cost of the mIRA EDSS, and the training costs incurred to facilitate the integrated management of patients by healthcare providers. Healthcare resource use will also change if the intervention is successful. While for some women, improved management will avoid some future costs, healthcare resources used by others will increase as a consequence of receiving care that they would not have received in the absence of the intervention. Thus, the net impact might be to either increase or decrease costs. The improved health outcomes will be estimated using trial data on the number of ANC components received by women; the proportion of women identified with GDM or PIH; the proportion of women with GDM or PIH whose blood sugar and pressure are controlled to recommended targets (in both the control and intervention arms). This information will be combined with data from the literature to estimate the additional QALYs generated by the intervention. The final step is to combine the changes in resource use and the changes in health outcomes in a single measure that can potentially be compared with the cost-effectiveness of other interventions for this patient group, or other patient groups.

### Data management

There will be two sources of data– the mIRA EDSS and the data collected by the field research staff and entered into the Microsoft Access template. All electronic data will be encrypted, password-protected, and stored in secure servers and computer networks.

The project’s Data Manager will export data from the cloud-based server and validate the dataset at regular intervals to check any possible data inconsistencies. All data management activities like data cleaning and data merging will be done by using the statistical software Stata. Any data related queries will be shared with the project team in a Query Management Excel Sheet. For any additional surveys (like Facility Survey or MCP Card reports), the Data Manager will create a data entry application in Microsoft Access or REDCap software. The Data Manager will also create informative dashboards and data reports using data visualization tools like Microsoft PowerBI and BI-tools for Excel. Access to study documents and data will be granted to selected members of the research team as per PHFI’s data sharing policy. All study data will only be identified by a participant study ID number, and medical confidentiality will be preserved in accordance with International Conference on Harmonisation (ICH) Good Clinical Practice (GCP) guidelines. Any notes with personal identifiers will only be accessible to the research staff that are in contact with pregnant women, those responsible for coordinating visits, and study personnel authorized by the principal investigators. At the end of the study, the electronic database will be archived in accordance with institutional procedures for 10 years.

### Data monitoring

The research staff will compile data collected throughout the trial to prepare monitoring reports. These reports will include data on recruitments per site, dropouts, details from intervention monitoring, intervention usage rate, adverse events and serious adverse events reported. These will be shared by the operational group comprised of the study research staff with the Trial Steering Committee and the Independent Data Monitoring Committee to ensure that the trial is progressing according to the planned objectives and milestones. These committees include external members, independent of the sponsors and funders, as well as the collaborating institutions, who meet annually. The Trial Steering Committee has been formed with aim to provide oversight for the trial. The Trial Steering Committee members are expected to be constructively critical of the ongoing trial while also being supportive of its aims and methods. The Independent Data Monitoring Committee will monitor the data emerging from the trial, in particular as they relate to the safety of participants, and to advise the Trial Steering Committee on whether there are any reasons for the trial not to continue.

Monthly project monitoring reports, compiled and summarized by the operational group [37] will be shared with the Trial Management Group to facilitate the compilation of lessons learned. Key findings may also be discussed along with the progress updates shared in the annual meetings with the Trial Steering Committee and the Independent Data Monitoring Committee.

### Adverse events

The mIRA EDSS is not evaluating any new drug or invasive procedure that requires specific monitoring of safety parameters. Rather, it is a health-systems study that is advancing the implementation of existing, evidence-based guidelines for PIH, GDM, anaemia management, and routine ANC. Information about the occurrence of any adverse event will be sought at all scheduled visits/calls by the field research staff, and outside of scheduled visits by participant self‐report. Patients will have access to the physician (MO) at the PHCs for remedial measures in the event of any adverse events. The healthcare providers reserve the right to reject any recommendations suggested by the mIRA EDSS, if not considered suitable for the patient. Reporting of all serious adverse events will be done to the International Data Monitoring Commitee in a biannual report. The clinical adverse events reported will include event of maternal intensive care unit (ICU) admission, maternal death in pregnancy, uncontrolled blood sugar, and maternal ketoacidosis due to hyperglycemia. Apart from the clinical adverse events, we will also follow Food and Drug Administration’s (FDA) guide to classify other non-clinical adverse events into three categories: (1) errors of commission, such as accessing the wrong patient’s record or overwriting one patient’s information with another’s; (2) errors of omission of transmission, such as the loss or corruption of vital patient data; (3) errors in data analysis, including medication dosing errors of several orders of magnitude [38].

## Discussion

This paper describes the protocol for a cluster-randomized controlled trial to evaluate the effectiveness of an mHealth intervention (i.e., the mIRA EDSS) in enhancing the quality of ANC and improving the screening and management of PIH, GDM, and anaemia, in rural primary healthcare settings in India. To the best of our knowledge, only a few mHealth interventions in India have been tested for improving maternal and child health outcomes [22–26]. Of these, the two mHealth interventions [24, 25] that involved a clinical decision-support system only targeted community health workers who worked primarily at the SC level and were responsible for referring pregnant women to the PHC. However, the results from these studies are not yet available.

To make a significant impact on improving maternal and child health, there is a need for an intervention that can seamlessly work at multiple levels of the health system, can be used by different cadres of health care providers and has the potential to be integrated within the existing clinic work flow. We address these important aspects in the current study as we plan on testing the mIRA EDSS which works at two facility levels (PHCs and SC), is also customized for use by three cadres of healthcare of providers – physicians (MOs), staff nurses, and ANMs and can be easily integrated on a “plug and play” mode within the existing clinic work flow and digital infrastructure.

Interventions work best when tailored to the settings and cultural context. A unique feature of the mIRA EDSS is its development process. It was co-designed by meaningfully engaging relevant subject experts (international and national), regional and local stakeholders (state and district health officials), and its targeted users (three cadres of healthcare providers – ANM, staff nurse, and MO). Further, the intervention development has also been informed by a robust formative research process in 23 health facilities. This co-design approach has helped in ensuring that the evidence-based screening and management recommendations produced are tailored to the state’s as well as the healthcare provider’s needs, and customized as per the local digital infrastructure at the facilities to increase the likelihood for a wider scale-up, if it is proved to be effective.

Furthermore, during the implementation, health officials will be involved in the supervision and management of the work at the trial facilities. This will also help in a smooth transition during potential scale-up, if the mIRA EDSS is effective. Another strength of this study is that the team assembled to develop the intervention and conduct the trial has experience in designing and successfully implementing similar mHealth interventions for non-pregnant populations. The committees involved in forming the guidelines for quality ANC, and screening and management of PIH, GDM, and anaemia included national and international subject experts in the respective areas, ensuring that the most current evidence was used.

If the mIRA EDSS is effective in enhancing the quality of ANC, the insights that India will gain into the feasibility and cost of implementing an EDDS, through the robust process evaluation and economic evaluation, respectively, may inform potential national scale-up. This scale-up can be facilitated with the increasing advances in information technology and would align well with India’s Digital Health Mission initiative. The lack of high-quality evidence on the efficacy, feasibility, and cost-effectiveness of mHealth interventions has been a barrier to the scaling of mHealth interventions in LMICs [39–42]. This trial, through its robust process and economic evaluation, will help answer these critical questions about the feasibility and cost-effectiveness of implementing mHealth interventions to enhance ANC. Lessons learned from this trial will also inform recommendations for designing, evaluating and scaling up similar mHealth interventions in other LMICs. The integration of the mIRA EDSS with other existing digital records will be useful in decreasing the record-keeping workload for healthcare providers. Furthermore, the feasibility to add further components to the mIRA EDSS like postpartum care, immunization, etc. can be explored.

This trial will provide much needed evidence on the effectiveness of mHealth interventions in improving the provision of ANC at the primary healthcare level. One possible limitation of the study would be ensuring compliance with the intervention. Measures will be taken to tackle this during the intervention lead-in period. During the training sessions and each monitoring visit, the field research staff will keep notes on major technical difficulties reported by the healthcare providers which might keep them from using the mIRA EDSS. Secondly, this trial will not measure improvement in health outcomes for the mother and the newborn. However, the possibility to access the state’s district-level data on these outcomes can be explored to conduct a secondary analysis for assessing whether there is an association with health outcomes. Thirdly, the findings from this study will not be generalizable to urban settings where many pregnant women seek care in private clinics and hospitals. 

## Supplementary Information

**Additional file 1.** 

**Additional file 2.** 

**Additional file 3.**

## Data Availability

Not applicable.

## Abbreviations

ANC: Antenatal Care
PIH: Pregnancy Induced Hypertension
GDM: Gestational Diabetes Mellitus
EDSS: Electronic Decision-Support System
PHC: Primary Health Centre
SC: Sub-centre
MO: Medical Officer
HMIS: Health Management Information Systems
