# Supplementary material for: A cluster randomized controlled trial of an electronic decision-support system to enhance antenatal care services in pregnancy at primary healthcare level in Telangana, India: trial protocol
Source: BMC Pregnancy Childbirth. 2023 Jan 26;23:72. doi: 10.1186/s12884-022-05249-y (PMC9878774; doi:10.1186/s12884-022-05249-y)
Supplement: Supplementary file 1 — Additional file 1. [file 12884_2022_5249_MOESM1_ESM.docx]

**Evaluation of the mIRA antenatal electronic decision support system**

**Participant Information Sheet (PIS) for pregnant women**

**Study title:** A cluster randomized trial of an mHealth integrated model of hypertension, diabetes and antenatal care in primary care settings in India and Nepal

**Simplified Study Title:** *Evaluation of the mIRA antenatal electronic decision support system*

**Name of PI(s):** Prof. Dorairaj Prabhakaran & Prof. Oona Campbell

**PI’s contact address:** Public Health Foundation of India (PHFI), Sector 44, Gurugram, Haryana-122002, India

**Introduction**

We would like to invite you to take part in a research study. Joining the study is entirely up to you. Before you decide, you need to understand why the research is being done and what it would involve. One of our team will go through this information sheet with you, and answer any questions you may have. Ask questions if anything you read is not clear or if you would like more information. Please feel free to talk to others about the study if you wish. Take time to decide whether or not to take part.

**What is the purpose of the study?**

Public Health Foundation India (PHFI), Kathmandu University and The London School of Hygiene and Tropical Medicine (LSHTM) developed an electronic decision support system (EDSS, the ‘mIRA’ intervention) to improve the quality of antenatal care in primary health facilities. The EDSS is tablet-based and is used by healthcare providers during antenatal care visits to record clinical information about their patients. As part of this study, some facilities are using the EDSS and some are not. The purpose of this study is to understand if and how the EDSS improves the quality of antenatal care in India and Nepal.

**Why have I been asked to take part?**

You are attending the facility today for an antenatal visit, are over age 18 and less than 29 weeks pregnant.

**Do I have to take part?**

No, you do not have to take part. It is your choice. If you choose not to take part, you will continue to receive all the services as normal.

**What will happen to me if I take part?**

If you decide to take part, we will ask you to complete and sign the consent form to observe your antenatal visit today and your next routine visit and to access your Mother and Child Protection (MCP) card. The fieldworker will also ask for your contact information to coordinate your next visit.

**What will I have to do?**

If you take part, you do not have do anything besides sign the consent form.

**What are the possible risks and disadvantages?**

Talking to the fieldworker and signing the consent form will take some of your time as you wait to be seen today. You may sometimes feel uncomfortable with having your visit and check-up with the doctor, nurse or midwife observed. If this happens, you may ask the fieldworker to stop the observation or to leave the room at any time, but still we hope you will take part in the study.

**What are the possible benefits?**

We cannot promise that you will benefit from being in the study. The information we get from the study will help our knowledge and understanding of how to improve the delivery of antenatal care in India and Nepal.

**What if something goes wrong?**

If you have a concern about any aspect of this study, you can speak to the researchers who will do their best to answer your questions (contact details below). If you remain unhappy and wish to complain formally, you can do this by contacting Monica Chaudhry (details below).

**Can I change my mind about taking part?**

Yes. You can leave the study at any time by telling the fieldworker that you no longer wish to have your antenatal visit observed or by contacting the researchers (details below). You do not have to give a reason for wanting to leave the study. Leaving the study will not affect the services that you receive nor will it have any other repercussions. However, in alignment with the ‘National Ethical Guidelines for Biomedical and Health Research Involving Human Participants’ from the Indian Council of Medical Research (ICMR), the data obtained till the time of your withdrawal will be used for analysis as per the study design. Details on how we will ensure confidentiality of the collected data has been covered below.

**What will happen to information collected about me?**

We will use information from your antenatal visit and records for this research project. All information collected about you will be kept confidential. Only the study staff and authorities who check that the study is being carried out properly will be allowed to look at information about you. We will keep all information about you safe and secure.

Information about you will be de-identified. This means that any information about you that leaves the facility will have your name and address removed so that you cannot be recognised and your data will have a code number instead.

Your personal details, meaning your name and other identifiable information, will be kept in a different safe place to the other study information and will be destroyed at the end of the study. At the end of the project, the study data will be archived for 10 years. The data will be placed in a data repository and made available to other researchers worldwide for research and to improve medical knowledge and patient care. Your personal information will not be included and there is no way that you can be identified.

**What are your choices about how your information is used?**

You can stop being part of the study at any time, without giving a reason.

**Will I get any compensation or reimbursement for participating?**

We will keep you updated on the reports from the blood and urine tests, conducted as a part of your routine pregnancy care along with relevant advice. In case any abnormality is detected, you will be contacted and informed about the same immediately. We will not be able to provide any monetary compensation to you for participating in this study.

**What will happen to the results of this study?**

The study results will be published in a medical journal so that others can learn from them. Your personal information will not be included in the study report and there is no way that you can be identified from it.

**Who is organising and funding this study?**

Public Health Foundation India is the sponsor of the research and they have full responsibility for the project including the collection, storage and analysis of your data, and will act as the Data Controller for the study. This means that we are responsible for looking after your information and using it properly. The study is funded by the Medical Research Council, United Kingdom and the Department of Biotechnology, India.

**Who has reviewed this study?**

All research involving human participants is looked at by an independent group of people, called a Research Ethics Committee, to protect your interests. This study has been reviewed and approved by The London School of Hygiene and Tropical Medicine Research Ethics Committee, the Public Health Foundation of India (PHFI) and the Institutional Review Committee of Kathmandu University School of Medical Sciences.

**Further information and contact details**

Thank you for taking time to read this information sheet. If you think you will take part in the study please read and sign the consent form.

If you would like any further information, please use the contact details provided below who can answer any questions you may have about the study.

Contact details:

XXXXXX XXXXXXXX

Public Health Foundation of India

Phone no. xxxxxxxxx

Email: [xxxxxxxxxx@phfi.org](mailto:xxxxxxxxxx@phfi.org)

Your signature on consent form means that you understand the information given to you about the study. If you sign the form it means that you agree to join the study.
