# Supplementary material for: A cluster randomized controlled trial of an electronic decision-support system to enhance antenatal care services in pregnancy at primary healthcare level in Telangana, India: trial protocol
Source: BMC Pregnancy Childbirth. 2023 Jan 26;23:72. doi: 10.1186/s12884-022-05249-y (PMC9878774; doi:10.1186/s12884-022-05249-y)
Supplement: Supplementary file 2 — Additional file 2. [file 12884_2022_5249_MOESM2_ESM.docx]

**Evaluation of the mIRA antenatal electronic decision support system**

**Informed Consent Form (ICF) for Pregnant Women**

**Study Title:** A cluster randomized trial of an mHealth integrated model of hypertension, diabetes and antenatal care in primary care settings in India and Nepal

**Name of PI(s):** Prof. Dorairaj Prabhakaran & Prof. Oona Campbell

| **Statement** | **Please tick each box** |
| --- | --- |
| I confirm that I have read the information sheet dated Jun 14 2021 (version 3) for the above named study. I have had the opportunity to consider the information, ask questions and have these answered satisfactorily. |  |
| I understand that my participation is voluntary and that I can withdraw at any time. |  |
| I understand that relevant sections of my clinic records and information collected during the study may be looked at by researchers directly involved in this study. I give permission for these individuals to access to my records. |  |
| I agree that my interview will be audio recorded and interviewer will also take some notes. |  |
| I understand that this information may be shared via a public data repository or by sharing directly with other researchers, and that I will not be identifiable from this information. |  |
| I understand that a fieldworker will observe my visit today and my next routine visit. |  |
| I agree to take part in this study. |  |

|  |  |  |  |  |
| --- | --- | --- | --- | --- |
| Name of Participant (print) |  | Signature |  | Date |
|  |  |  |  |  |
| Name of third party witness |  | Signature |  | Date |
| **Statement by person taking consent**  **I** confirm that the participant was given an opportunity to ask questions about the study, and all the questions asked by the participant have been answered correctly and to the best of my ability. I confirm that the individual has not been coerced into giving consent, and the consent has been given freely and voluntarily. A copy of the consent form has been given to the participant. | | | | |
|  |  |  |  |  |
| Name of person taking consent (print) |  | Signature |  | Date |
