# Supplementary material for: A cluster randomized controlled trial of an electronic decision-support system to enhance antenatal care services in pregnancy at primary healthcare level in Telangana, India: trial protocol
Source: BMC Pregnancy Childbirth. 2023 Jan 26;23:72. doi: 10.1186/s12884-022-05249-y (PMC9878774; doi:10.1186/s12884-022-05249-y)
Supplement: Supplementary file 3 — Additional file 3. [file 12884_2022_5249_MOESM3_ESM.docx]

# ­­ANC OBSERVATION CHECKLIST

***INFORMED CONSENT****: Proceed with this tool only* ***after*** *the participant provides written informed consent.*

***PHOTOS OF PARTICIPANT’S DOCUMENTS:*** ***After*** *observing the ANC consultation, please photograph the first three pages of the mother and child protection card (MCP) card, OPD slip, investigation records and any other documents the woman brought to the ANC consultation. You must cover the woman’s name when taking photos of these and save the files with the woman’s study ID number.*

| **Date** | / / |
| --- | --- |
| **Start time for ANC consultation** | : AM /PM |
| **End time for ANC consultation** | : AM /PM |

| **Staff ID and signature of the person completing this form**   \|  \|  \|  \|  \| \| --- \| --- \| --- \| --- \|   Sign: |  |
| --- | --- | --- | --- | --- | --- |
| **Staff ID and signature of the person certifying this form**   \|  \|  \|  \|  \| \| --- \| --- \| --- \| --- \|   Sign: | Date of certifying:   \| D \| D \| / \| M \| M \| / \| Y \| Y \| \| --- \| --- \| --- \| --- \| --- \| --- \| --- \| --- \| |

| **Record keeping** | | | |
| --- | --- | --- | --- |
| **How was the participant’s information recorded during the consultation and who recorded it?** | | | |
| **MCP card** | During the examination | After examination, before woman left the facility | After woman left the facility |
|  | \|  \| ANM \| \| --- \| --- \| \|  \| Staff Nurse \| \|  \| MO \| \|  \| DEO \| | \|  \| ANM \| \| --- \| --- \| \|  \| Staff Nurse \| \|  \| MO \| \|  \| DEO \| | \|  \| ANM \| \| --- \| --- \| \|  \| Staff Nurse \| \|  \| MO \| \|  \| DEO \| |
| **mIRA EDSS** | During the examination | After examination, before woman left the facility | After woman left the facility |
|  | \|  \| ANM \| \| --- \| --- \| \|  \| Staff Nurse \| \|  \| MO \| \|  \| DEO \| | \|  \| ANM \| \| --- \| --- \| \|  \| Staff Nurse \| \|  \| MO \| \|  \| DEO \| | \|  \| ANM \| \| --- \| --- \| \|  \| Staff Nurse \| \|  \| MO \| \|  \| DEO \| |

| **Section 1: Symptoms** | | | **If yes, did the woman report having the symptom?**  **Tick ☑ ‘Yes’, or ‘No’, as applicable**   \| Yes \|  \| No \| \| --- \| --- \| --- \| | **If yes, what the provider do?**  A = No response/action  B = Talked/enquired and discussed the problem  C = Gave advice/counselling  D = Prescribed medicine to resolve the problem  E = Asked the woman to get a laboratory test  F = Referred to another facility  **Enter the applicable code in the provided box (Separate letters by a comma “,” if multiple actions were performed).** |
| --- | --- | --- | --- | --- | --- | --- | --- |
| **Were the following symptoms discussed (either by the provider asking or the woman mentioning)?**   \| Yes \|  \| No \| \| --- \| --- \| --- \| | | |  |  |
| 1.1 | Nausea |  | \|  \|  \|  \| \| --- \| --- \| --- \| | \| Action \|  \| If others, Specify……………………. \| \| --- \| --- \| --- \| |
| 1.2 | Vomiting | \|  \|  \|  \| \| --- \| --- \| --- \|  \|  \|  \|  \| \| --- \| --- \| --- \|  \|  \|  \|  \| \| --- \| --- \| --- \| | \|  \|  \|  \| \| --- \| --- \| --- \| | \| Action \|  \| If others, Specify……………………. \| \| --- \| --- \| --- \| |
| 1.3 | Vaginal bleeding |  | \|  \|  \|  \| \| --- \| --- \| --- \| | \| Action \|  \| If others, Specify……………………. \| \| --- \| --- \| --- \| |
| 1.4 | Severe headache | \|  \|  \|  \| \| --- \| --- \| --- \| | \|  \|  \|  \| \| --- \| --- \| --- \| | \| Action \|  \| If others, Specify……………………. \| \| --- \| --- \| --- \| |
| 1.5 | Decreased or absent foetal movement | \|  \|  \|  \| \| --- \| --- \| --- \|  \|  \|  \|  \| \| --- \| --- \| --- \| | \|  \|  \|  \| \| --- \| --- \| --- \| | \| Action \|  \| If others, Specify……………………. \| \| --- \| --- \| --- \| |
| 1.6 | Severe abdominal pain |  | \|  \|  \|  \| \| --- \| --- \| --- \| | \| Action \|  \| If others, Specify……………………. \| \| --- \| --- \| --- \| |
| 1.7 | Blurred vision |  | \|  \|  \|  \| \| --- \| --- \| --- \| | \| Action \|  \| If others, Specify……………………. \| \| --- \| --- \| --- \| |

|  |  |  |
| --- | --- | --- |

| **Section 2: Investigations** | | |
| --- | --- | --- |
| **Did the provider undertake the following procedures?** | | \| Yes \|  \| No \| \| --- \| --- \| --- \| |
| 2.1 | \|  \| \| --- \|  \|  \| \| --- \|   Blood pressure measurement and record it on MCP card | \|  \|  \|  \| \| --- \| --- \| --- \| |
| 2.2 | Urine dipstick test | \|  \|  \|  \| \| --- \| --- \| --- \| |
| 2.3 | Blood hemoglobin test | \|  \|  \|  \| \| --- \| --- \| --- \| |
| 2.4 | Blood glucose test  If yes, circle which: | \|  \|  \|  \| \| --- \| --- \| --- \| |
|  | *Complete blood count; Random blood sugar (RBS) by glucometer; Fasting blood sugar (FBS) by glucometer; Oral glucose test (OGTT) by capillary blood (with glucometer)* | |

| **Section 3: Diagnosis** | | | **If yes, did the provider tell the women she was at risk or had the condition:**   \| Yes \|  \| No \| \| --- \| --- \| --- \| | **If yes, what the provider do?**  A = No response/action  B = Talked/enquired and discussed the problem  C = Gave advice/counselling  D = Prescribed medicine to resolve the problem  E = Asked the woman to get a laboratory test (or additional laboratory tests)  F = Referred to another facility  **Enter the applicable code in the provided box (Separate letters by a comma “,” if multiple actions were performed).** |
| --- | --- | --- | --- | --- | --- | --- | --- |
| **Did the provider tell the women about what they found? (**Discussed the reading or test result with the woman) **Tick ☑ ‘Yes’, or ‘No’, as applicable**   \| Yes \|  \| No \| \| --- \| --- \| --- \| | | |  |  |
| 3.1 | PIH (high blood pressure, eclampsia)   \|  \|  \|  \| \| --- \| --- \| --- \|  \|  \|  \|  \| \| --- \| --- \| --- \| |  | \|  \|  \|  \| \| --- \| --- \| --- \| | \| Action \|  \| If others, Specify……………………. \| \| --- \| --- \| --- \| |
| 3.2 | GDM (high blood sugar) |  | \|  \|  \|  \| \| --- \| --- \| --- \| | \| Action \|  \| If others, Specify……………………. \| \| --- \| --- \| --- \| |
| 3.3 | Severe anaemia | \|  \|  \|  \| \| --- \| --- \| --- \| | \|  \|  \|  \| \| --- \| --- \| --- \| | \| Action \|  \| If others, Specify……………………. \| \| --- \| --- \| --- \| |

| **Section 4: Counselling** | | |
| --- | --- | --- |
| **Did the provider tell the participant that she should return for help if she experiences the following?:** | | \| Yes \|  \| No \| \| --- \| --- \| --- \| |
| 4.1 | Severe vomiting | \|  \|  \|  \| \| --- \| --- \| --- \| |
| 4.2 | Vaginal bleeding | \|  \|  \|  \| \| --- \| --- \| --- \| |
| 4.3 | Severe headaches | \|  \|  \|  \| \| --- \| --- \| --- \| |
| 4.4 | Decreased or no fetal movement | \|  \|  \|  \| \| --- \| --- \| --- \| |
| 4.5 | Severe abdominal pain | \|  \|  \|  \| \| --- \| --- \| --- \| |
| 4.6 | Blurred vision | \|  \|  \|  \| \| --- \| --- \| --- \| |
| **OTHER ASPECTS OF HEALTH** | | |
| 4.7 | Did the provider mention diet? | \|  \|  \|  \| \| --- \| --- \| --- \| |
| 4.8 | Did the provider ask the woman about her mental health | \|  \|  \|  \| \| --- \| --- \| --- \| |
| **OTHER AS PART OF PROCESS** | | |
| 4.9 | Did the provider mention when the woman should return for the next ANC visit? | \|  \|  \|  \| \| --- \| --- \| --- \| |
| 4.9.1 | Did the provider ask whether the woman had any other questions or queries? | \|  \|  \|  \| \| --- \| --- \| --- \| |
